# Features derived from blood pressure and intracranial pressure predict elevated intracranial pressure events in critically ill children

**DOI:** 10.1038/s41598-022-25169-3

**Published:** 2022-12-12

**Authors:** Kassi Ackerman, Akram Mohammed, Lokesh Chinthala, Robert L. Davis, Rishikesan Kamaleswaran, Nadeem I. Shafi

**Affiliations:** 1 University of Tennessee Health Science Center, Memphis, TN USA; 2 Emory University School of Medicine, Atlanta, GA USA; 3 Georgia Institute of Technology, Atlanta, GA USA

**Keywords:** Computational biology and bioinformatics, Neuroscience, Medical research, Paediatric research

## Abstract

Clinicians frequently observe hemodynamic changes preceding elevated intracranial pressure events. We employed a machine learning approach to identify novel and differentially expressed features associated with elevated intracranial pressure events in children with severe brain injuries. Statistical features from physiologic data streams were derived from non-overlapping 30-min analysis windows prior to 21 elevated intracranial pressure events; 200 records without elevated intracranial pressure events were used as controls. Ten Monte Carlo simulations with training/testing splits provided performance benchmarks for 4 machine learning approaches. XGBoost yielded the best performing predictive models. Shapley Additive Explanations analyses demonstrated that a majority of the top 20 contributing features consistently derived from blood pressure data streams up to 240 min prior to elevated intracranial events. The best performing prediction model was using the 30–60 min analysis window; for this model, the area under the receiver operating characteristic window using XGBoost was 0.82 (95% CI 0.81–0.83); the area under the precision-recall curve was 0.24 (95% CI 0.23–0.25), above the expected baseline of 0.1. We conclude that physiomarkers discernable by machine learning are concentrated within blood pressure and intracranial pressure data up to 4 h prior to elevated intracranial pressure events.

## Introduction

The importance of minimizing intracranial pressure (ICP) elevations in the setting of traumatic brain injury (TBI) has been underscored by several studies. For instance, one study demonstrated that the number of 5-min episodes of intracranial hypertension was predictive of poor outcome^1^; another study showed that for every hour that ICP was greater than 20, the odds of poor outcomes increased by 4.6%^2^.

Clinically recognizable, stereotyped vital sign changes (known as the Cushing reflex^3^) cannot be relied upon to predict elevations in ICP because they occur late and indicate impending herniation. Often, tachycardia due to an early sympathetic surge precedes an ominous bradycardia^4^, suggesting that hemodynamic signals are likely to contain features (or “physiomarkers”) which could be predictive of ICP elevation.

The application of machine-learning to physiologic signals offers an opportunity to detect higher-order features, i.e. those not discernable by human care providers. We analyzed continuous streaming data collected from bedside monitors of children with severe brain injuries using machine learning methods to evaluate (a) whether there are novel and differentially expressed physiomarkers of elevated ICP events contained within hemodynamic and other physiologic signals, and (b) if these physiomarkers can reveal robust predictive performance.

## Methods

### Clinical framework and data pre-processing

We conducted a retrospective study of children aged 2–17 years who were admitted to the Pediatric ICU and Neuro ICU of a tertiary/quaternary children’s medical center from October, 2017 until December, 2020. This study was approved, and informed consent was waived by the Institutional Review Board of the University of Tennessee Health Science Center (Memphis, TN). All methods were performed in accordance with the relevant guidelines and regulations.

To be included, children had to have undergone concurrent invasive arterial blood pressure (ABP) and ICP monitoring. Records which had < 4 h of concurrent electrocardiogram (EKG), ABP and ICP data were excluded.

**Figure 1:**
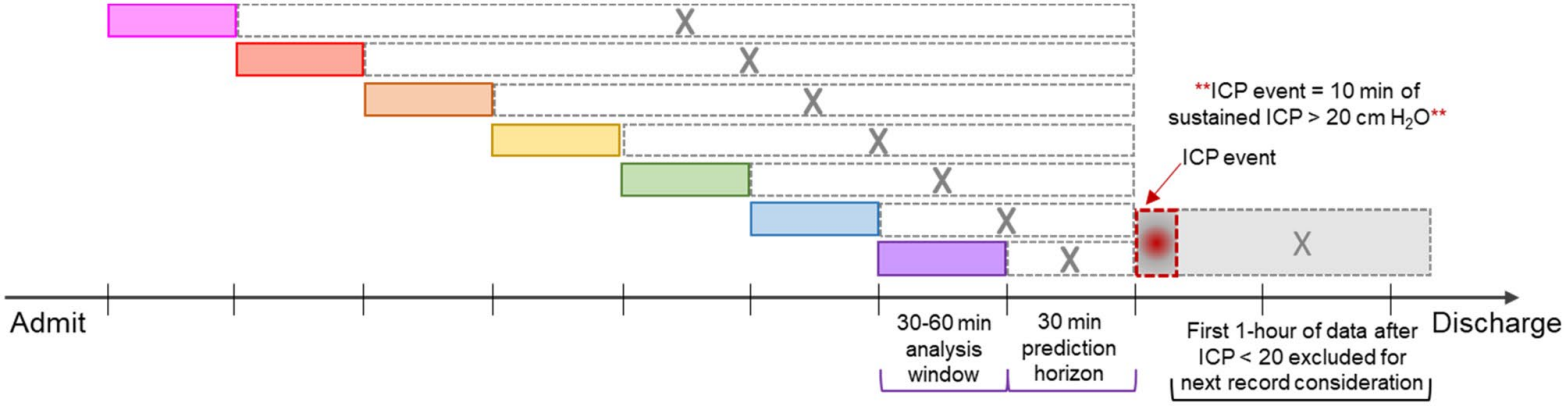
Elevated ICP event schematic. The prediction horizon is the time prior to the ICP event over which features from the preceding analysis window are used for prediction. ICP had to remain < 20 cm H_2_O for one hour before subsequent data could be considered for inclusion.

All patients were managed in accordance with standards of care for brain injury, including the pediatric TBI guidelines^5^. An elevated ICP event (case) was defined as ≥ 20 cm H_2_O for 10 min, and events were considered independent if they occurred at least 5 h apart in the same patient (Fig. 1). Control records were derived from patients without elevated ICPs as well as from those with cases when ICPs were < 20 cm H_2_O for 6 h. Thus, each patient could provide cases and controls.

Six physiologic signals (ABP, consisting of systolic, diastolic, and mean blood pressures; electrocardiogram (EKG)-derived heart rate (HR); pulse rate from plethysmography; ICP; cerebral perfusion pressure (CPP); and oxygen saturation) were each sampled at 1-min intervals via a Drager Monitoring system since this is a frequency of data acquisition that is common among many ICUs.

All records were manually adjudicated by an ICU physician to confirm criteria and identify signal artifact. Records with > 10% missing data or significant artifact burden in any of the SBP, DBP, MAP, or CPP data streams were excluded. Next, line graphs of the data were reviewed to manually identify obvious blood pressure artifacts. These were removed with a two-step artifact detection algorithm after the standard deviation (SD) of the entire 4-h record was determined. Because artifacts were most dramatically represented in the systolic blood pressure (SBP) data stream, any SBP value which exceeded the first datapoint of a 5-min block by two SDs was removed and forward-filled; this was repeated after sliding the 5-min block forwards by 1-min through the entire record. This step allowed us to identify and remove “wide artifact”. Next, any SBP minute-to-minute change that exceeded 30% was removed and forward-filled, allowing identification and removal of “narrow artifact” that was not captured in the first step. At the points of artifact, SBP, MAP, DBP, and CPP data streams were all corrected. An example of artifact removal is provided in supplementary figures.

### Machine learning

4-h records were divided into 8 non-overlapping but consecutive 30-min analysis windows as depicted in Fig. 1. Prediction horizons—that is, the times in advance each model bundle was asked to predict the elevated ICP event—were increased by 30 min until the beginning of the record. Eighteen statistical features were derived from each physiologic signal from the 30-min window immediately prior to each prediction horizon. These were mean, median, max, min, quantiles (0.2, 0.4, 0.6, 0.8), variance, standard deviation, aggregated variance, centroid, kurtosis, skew, sample entropy, binned entropy, absolute change, and mean change.

Predictive modeling was performed with logistic regression (LR), support vector machine (SVM), random forest (RF), and XGBoost (XGB). The records were divided 70/30 for training and testing, respectively. During model training, we used a subset of the training data for hyperparameter selection using Bayesian optimization^6^. Due to a significant imbalance between the numbers of case events (records with elevated ICP events) and control periods (records without elevated ICP events), one control was chosen for each case in a random fashion during training. Training and testing were iterated with ten different 70/30 splits at each time window following 10 Monte Carlo simulations. Explainability of models generated were assessed using averaged Shapley Additive Explanations (SHAP) scores from each simulation^7^. Model performance was assessed not only by area under the receiver operating characteristic (AUROC) curves, but also by area under the precision-recall (AUPRC) curves, which are more appropriate for imbalanced data sets. Sensitivity, specificity, positive and negative predictive values were also determined. An illustration of the architecture can be found in supplementary figures.

## Results

### Case events, control periods, and data preprocessing

**Figure 2:**
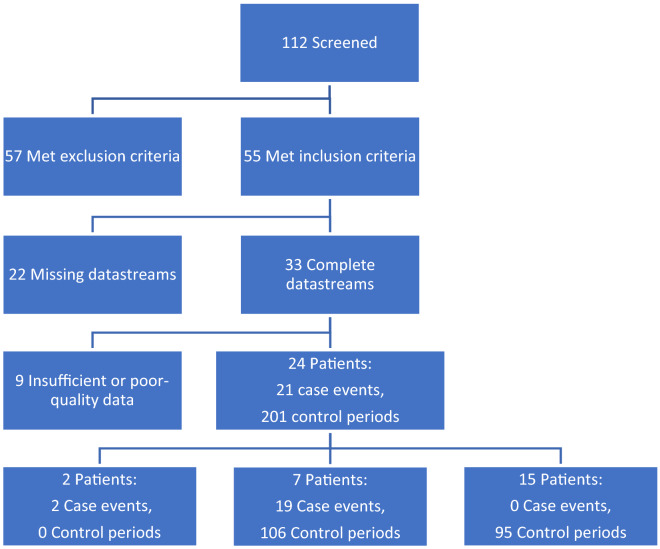
Consort diagram demonstrating the final data set. An elevated ICP event (≥ 20 cm H_2_O for 10 min) comprised a case event; events were considered independent if they occurred at least 5 h apart in the same patient. Control periods consisted of 6-h durations in which ICPs remained < 20 cm H_2_O. Thus, each patient could provide case events and control periods. Data was regarded as “insufficient” if > 20 min (~ 10% of a record) was of poor quality or missing.

112 patients were identified using hospital procedure records for external ventricular drains and intraparenchymal pressure monitoring devices placed at our institution. 57 of the 112 patients met exclusion criteria and 55 patients met inclusion criteria. Of the 55 patients, 33 had complete datasets. Reasons for missingness included patients being transported off unit, arterial line or sensor dysfunction, etc. 24 patients had quality data with limited artifact burden that satisfied definitions for elevated ICP events, control periods, or both. A total of 21 case events from 9 patients and 200 control periods from 22 patients were ultimately included for model development and testing (Fig. 2).

Our algorithmic artifact removal resulted in 1.2% of the data being replaced (means of 2.8 and 2.8 min replaced per 4-h record in cases and controls, respectively). 92% of the manually identified artifacts were removed.

Table 1 summarizes the clinical characteristics of the 24 patients who had elevated ICP events and those who did not. Supplemental Table 1 summarizes patient characteristics by elevated ICP events and control periods. Decompressive craniectomy was present during a higher percentage of control periods when compared to case events (71% and 48% respectively). Etiologies of brain injury were not dissimilar, although there were slightly more case events derived from patients with TBI, and slightly more control periods derived from patients with non-traumatic hemorrhage.

**Table 1:** Clinical characteristics of patients who had elevated ICP events vs patients who had no elevated ICP events.

	Patients (n = 24)	Patients with events (n = 9)	Patients with controls (n = 15)
Age (mean ± SD)	7 ± 5	6 ± 5	8 ± 5
Male (%)	12 (50%)	3 (33%)	9 (60%)
**Diagnostic category**			
Traumatic brain injury	16 (67%)	6 (67%)	10 (67%)
Non-traumatic hemorrhage	4 (17%)	1 (11%)	3 (20%)
Obstructive mass	3 (13%)	1 (11%)	2 (13%)
Ischemic^§^	1 (4%)	1 (11%)	0
Intraparenchymal pressure monitor (%)*	16 (67%)	7 (78%)	9 (60%)
Craniectomy (%)	16 (67%)	5 (56%)	10 (67%)

^§^Suffered a thrombotic stroke and cardiac arrest.

*Remainder had externalized ventricular drains (EVD). One patient had data from both an EVD and intraparenchymal pressure monitor but not simultaneously.

An intraparenchymal monitor was used equally and in the majority of patients; the remainder had an externalized ventricular drain, which were set to transduce continuously. Vasoactive support was less common during control periods (16%) compared to case events (29%), although mean vasoactive infusion scores (VIS) were similar (1 ± 4 and 3 ± 5, respectively). The remaining 43% of case events and 62% of control periods were not on anti-hypertensive infusions or vasoactive support. Pupil reactivity at the time of case and control records was similar. Deaths were distributed equally (1 case patient only, 1 control patient only, 1 patient contributing a case and control).

### Predictive modeling

**Figure 3:**
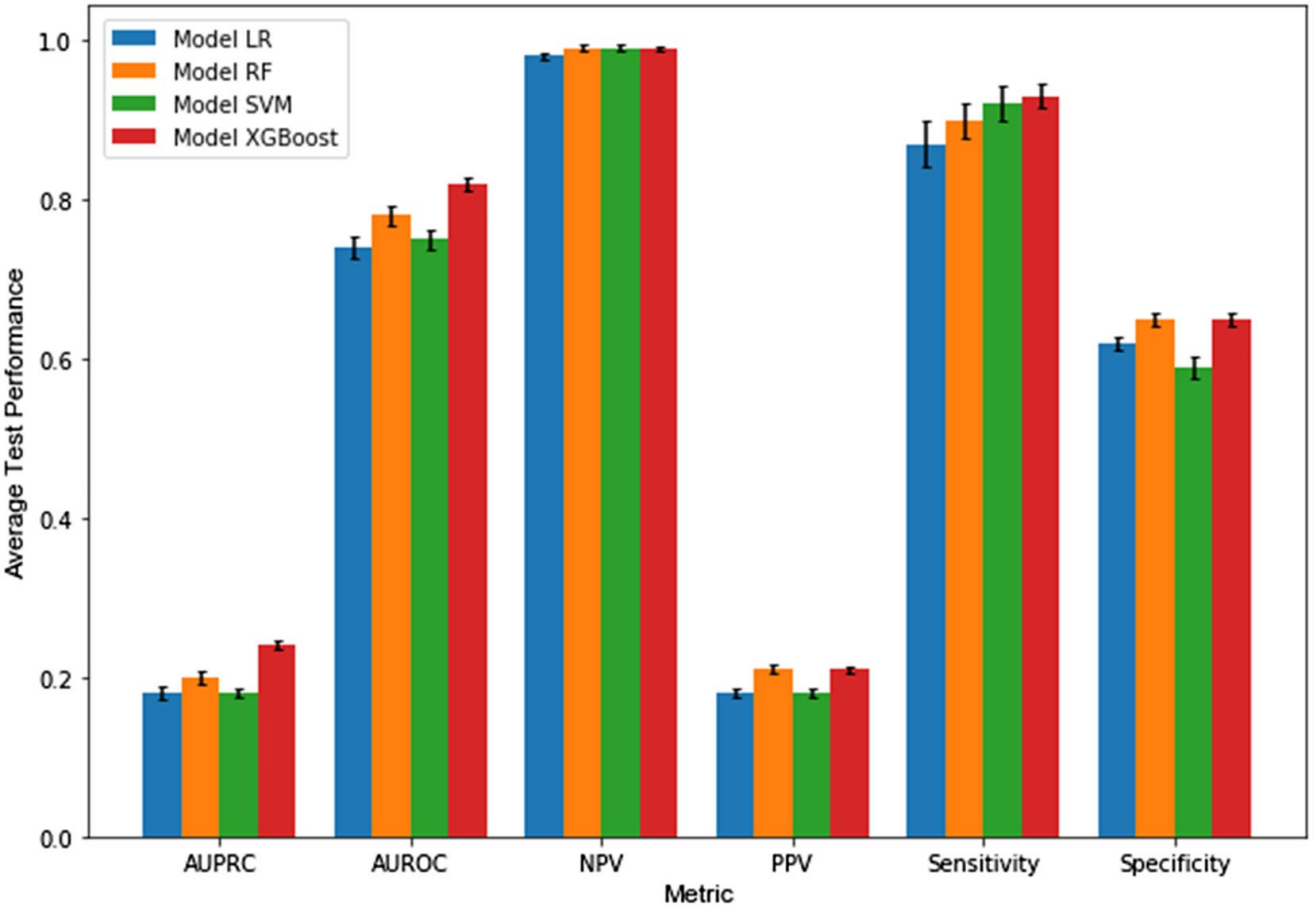
Performance of each modeling approach for predicting elevated ICP events with a 30 min prediction horizon. Error bars = 95% confidence intervals.

The relative performances for each of our modeling approaches at predicting elevated ICP events with a 30 min prediction horizon are depicted in Fig. 3. At the 30–60 min analysis window (i.e. 30 min prediction horizon), XGB produced a model benchmark AUROC of 0.82 (95% CI 0.81–0.83). The AUPRC benchmark at the 30 min horizon was 0.24 (95% CI 0.23–0.25), which was significantly above the baseline expected AUPRC of 0.10. Sensitivities of the models ranged from 0.87 to 0.93, with XGB achieving a sensitivity of 0.93 (95% CI 0.91–0.95). Specificities ranged from 0.59 to 0.65, with XGB achieving 0.65 (95% CI 0.64–0.66). Positive (PPV) and negative predictive values (NPV) for XGB were 0.21 and 0.99, respectively. We suspect these over-estimate real-world PPV and NPV since elevated ICP events likely have a lower prevalence than the 10:1 ratio utilized in our modeling. When we trained and tested the models using features derived from blood pressure alone, performance metrics dropped (see Supplemental Fig. 3).

### Feature expression in physiologic signals

**Figure 4:**
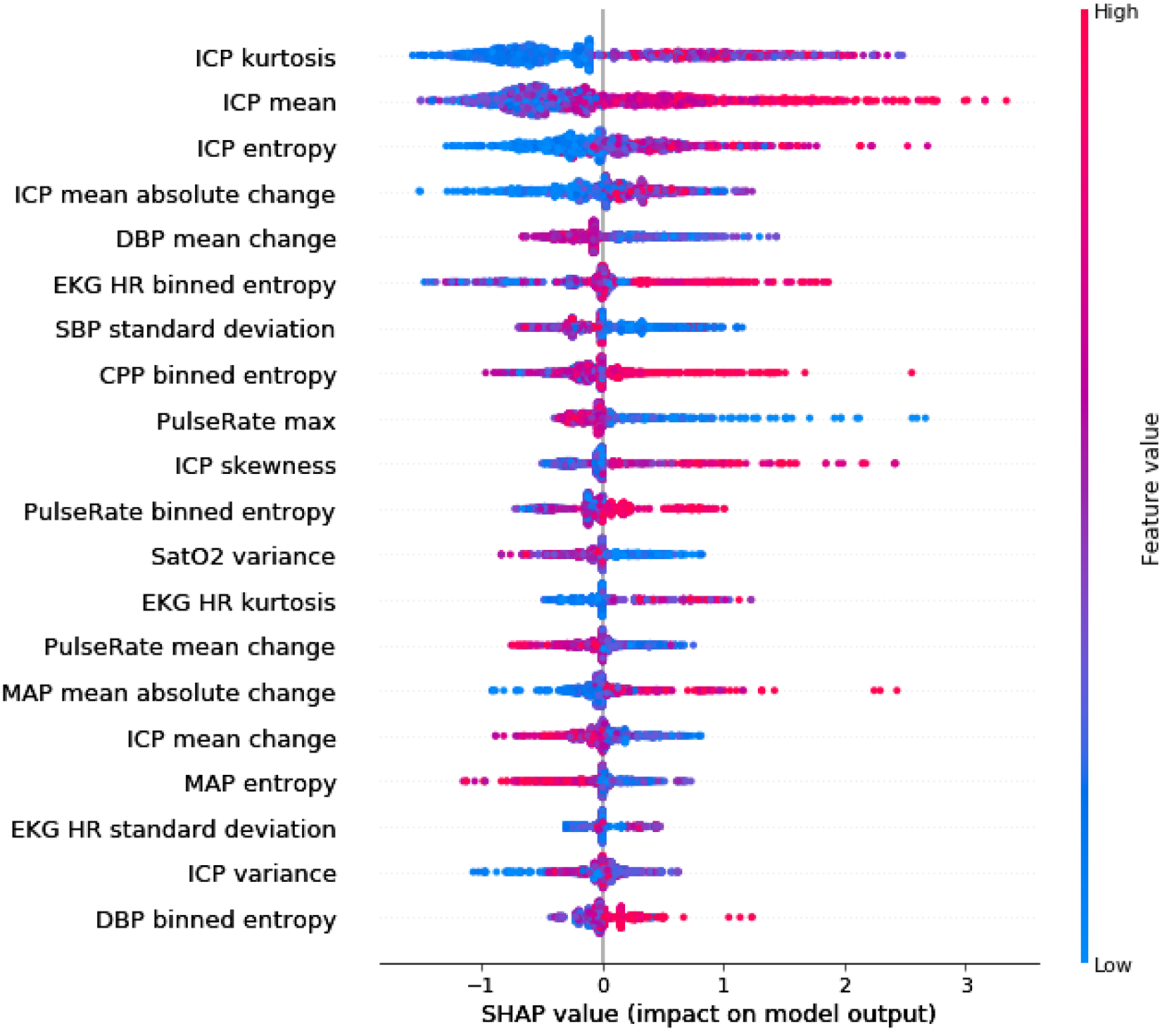
Average SHAP value plot, which assesses feature importance. Displayed in descending order of importance are the 20 features which were most contributory to the XGB model bundle performance with the 30-min prediction horizon, along with averaged SHAP values. For instance, low values (blue) of ICP kurtosis favor a prediction of “control” (negative SHAP values), whereas high values (red) of ICP mean favor a prediction of “event” (positive SHAP values).

The averaged SHAP value plot depicts how the top 20 features contributed to the performance of the XBG 30–60 min predictive model bundle (Fig. 4). For instance, lower values in ICP kurtosis of the fast-Fourier transform were associated with a lower likelihood—and higher average means in the ICP were strongly associated with a higher likelihood—of developing elevated ICPs. Higher dynamics and variability in both the ICP (sample entropy) and EKG (binned entropy) signals increased likelihood of elevated ICP events; conversely, lower dynamics and variability in DBP (mean change), SBP (standard deviation) and MAP (sample entropy) were associated with future elevated ICP events. SHAP value analysis after training and testing our models with blood pressure features alone can be seen in Supplemental Fig. 4. High/low values were less segregated to either side of 0, suggesting reduced directional prediction.

**Figure 5:**
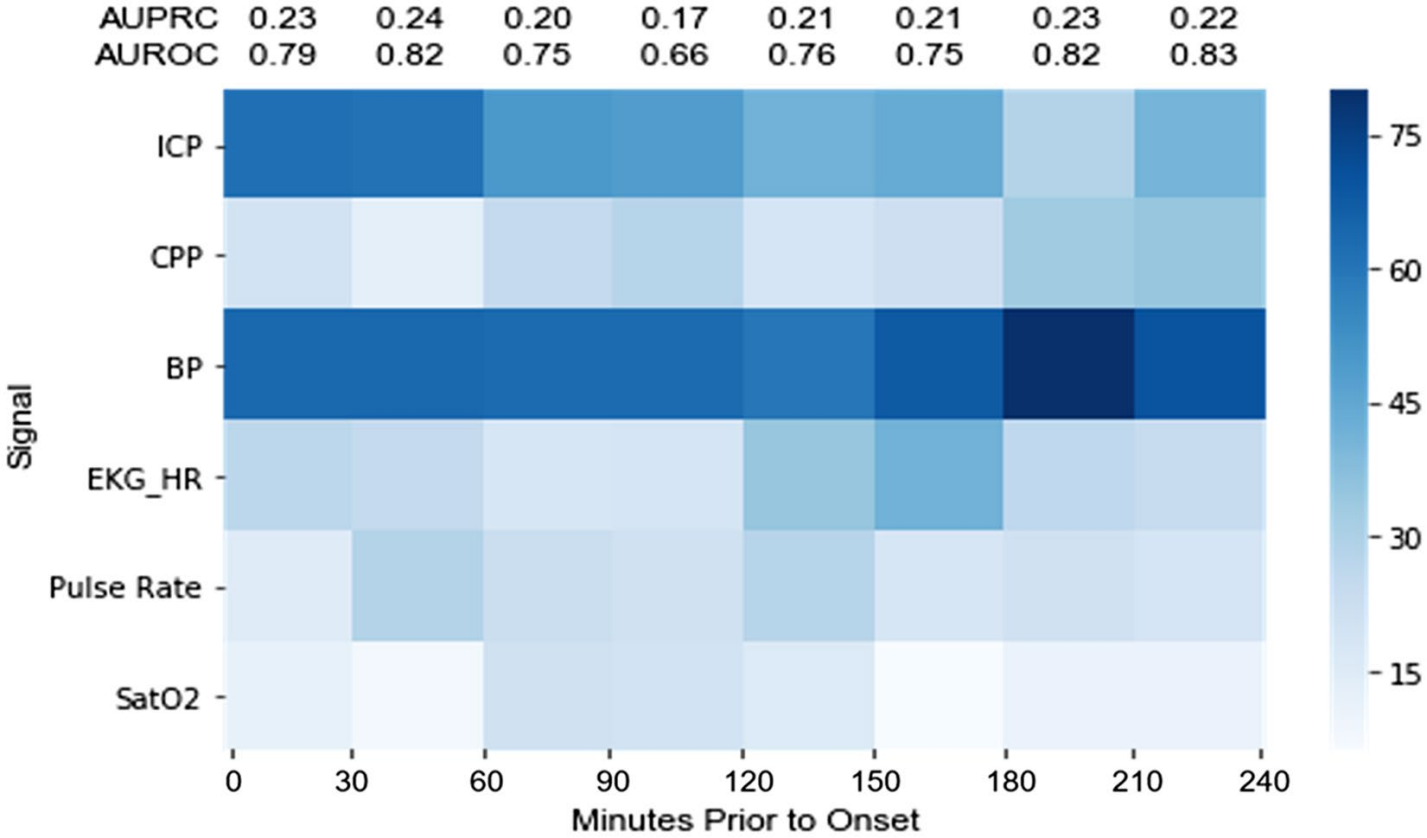
Heat map of most important features across 10 training–testing iterations in every 30 min analysis window. 200 features in each column are categorized by physiologic signal. Darker blue means a greater number of features derived from the physiologic signal.

Considering the 20 most contributing features in each of 10 Monte Carlo simulations with iterative splits, the heat map in Fig. 5 represents how many of these features derived from each physiologic signal in every 30-min analysis window. Said another way, this heat map conveys to what extent the top contributing features from each physiologic signal were differentially expressed.

Two results suggest the internal validity of this representation. First, differentially expressed features contained within the ICP signal increased progressively in time windows closer to elevated ICP events. This is an expected result and can be thought of as analogous to a ‘positive control’ as well as to the demonstration of ‘dose–response.’ Second, the plethysmography oxygen saturation (SatO2) signal contributed the fewest top 20 features across all time windows—again, this was an expected result, likening it to a ‘negative control’.

Strikingly, features contained within the blood pressure signal were the most differentially expressed across every time window up to 4-h prior to elevated ICP events, even matching ICP signal-derived features at 0–60 min. Features from within the blood pressure signal at 30–60 min derived 40%, 40%, and 20% from the systolic, diastolic, and mean blood pressures, respectively (not shown).

Heart rate derived features demonstrated modest differential expression.

## Discussion

The primary purpose of this pilot study was to determine whether novel physiomarkers for elevated ICP events can be detected using machine learning methods in the physiologic data streams from continuous bedside monitors of children with severe brain injury. Secondarily, we benchmarked the predictive performance of the modeling approaches we used.

Though we expected features derived from the heart rate signal to have high differential expression, we did not find this to be the case (Fig. 5). We found, instead, that features contained within the blood pressure signal were consistently the most differentially expressed, and that models trained on features derived from these in conjunction with ICP features performed best. This suggests that (a) non-ICP derived physiomarkers for elevated ICP events are derived predominantly from the blood pressure signal, and (b) ICP- and non-ICP derived physiomarkers are accessible to machine learning analyses.

Our findings are consistent with observations of both clinical and experimental biology. Hemodynamic changes routinely occur prior to elevated ICP events, which are thought to be regulated by the sympathetic nervous system with the aim of reducing ICP by autoregulatory cerebral vasoconstriction^4,8^; in fact, the “sympathetic surge” is regarded by clinicians as a harbinger of herniation events^9–12^. A relationship between ICP and sympathetic tone has also been demonstrated experimentally. In mice and humans, ICPs above 10 mm Hg resulted in elevations in renal and muscle sympathetic outflow, respectively^13^. A 10 mm Hg increase in ICP also caused renal sympathetic outflow-mediated BP elevations without changes in HR in sheep^14^. Notably, the strong contribution of blood pressure features to our XGB predictive model endured across every time window we tested (Fig. 5) as did our model performance (see AUPRC values), raising the provocative possibility that autonomic nervous system adaptation or dysfunction may produce predictive physiomarkers long before elevated ICP events occur, which we defined as over 20 cm H_2_O (~ 15 mmHg).

We do not believe this result can be causatively explained by clinical differences among case events and control periods. Children of different ages can have significantly disparate hemodynamic parameters, but the ages of case and control records were not different. Although 20% more control records were derived from patients who were not on either vasoactive or anti-hypertensive infusions, the mean VIS between case events and control periods was similar. That said, we cannot predict how vasoactive/inotropic agents, antihypertensives, or hemodynamic-influencing sedatives impact these physiomarkers. Also, despite more control records coming from patients with craniectomies, this surgery would only serve to reduce the chances of having elevated ICP events and not, in itself, impact hemodynamics.

The limited contribution of MAP from among the hemodynamic signals stands in interesting contrast to the findings of Güiza et al*.*, whose model predicted elevated ICP events 30 min prior to occurrence with an AUROC of 0.87 in an initial study^15^ using only ICP and MAP as model inputs. With a pediatric external validation cohort^16^, these authors found a lower AUROC of 0.79, comparable to our benchmark AUROC of 0.82. This may have been because their initial development cohort contained only 13 pediatric patients. Sensitivities were similar (92% and 93%), though our model achieved better specificity (48% vs 65%). Güiza et al.’s excellent predictive performance using MAP and ICP alone may be a reflection of their large training cohorts as well as their predominantly adult population which has less vital sign variation than children. It is also possible that different machine learning modeling approaches—and importantly, the features which are examined—may modulate the measured contribution of each physiologic signal. Nevertheless, our findings that differentially expressed physiomarkers are concentrated within the blood pressure signal as well as the ICP signal are consistent with Güiza et al*.*’s modeling results.

Conclusions from our small, pilot study of children with varied ages and severe brain injury pathologies derive from 24 patients, of which 9 had events, which is its principal limitation. Our use of multiple splits and Monte Carlo simulations across elevated ICP events and controls is an attempt to overcome this limitation as well as to correct for event-control imbalance. A more ideal cohort size would allow an equal number of events and controls to be derived from every patient, or alternatively, would have each patient contribute only one event or control period in a randomized fashion to allow splits across patients instead of across samples. Therefore, our results require external validation in a larger cohort, which will likely require multi-site collaboration.

Minute-to-minute data resolution may have constrained our model performance. Measures of change and variability were limited in our feature selection, and our design did not consider changes in feature values over time across analysis windows. Our records were curated and imbalanced. We did not study the impact of vasoactive agents or sedatives (e.g. propofol, dexmedetomidine) which may have hemodynamic effects, or of management conditions (e.g. craniectomy, type of ICP monitor, etc.), though their use appear similar across case events and control periods. Automated signal artifact removal algorithms would be required for any future bedside implementation.

In summary, we find that discriminatory physiomarkers discernable by machine learning methods are concentrated within blood pressure and ICP data for up to 4 h prior to future elevated ICP events in children with severe brain injuries, and models using ICP- and blood pressure-derived features demonstrate strong benchmark performance. Taken in aggregate, our findings support the idea that future attempts at high fidelity predictive modeling of elevated ICP events should leverage features contained within hemodynamic and ICP signals.

## Supplementary Information

Supplementary Information.

## Data Availability

The data supporting this study's findings are from the Methodist Le Bonheur Healthcare system and are not publicly available. Data are, however, available from the authors upon reasonable request and with permission of the Institutional Review Boards of the University of Tennessee Health Science Center and Methodist Le Bonheur Children's Hospital. To request data, please contact Dr. Nadeem Shafi by email at nshafi@uthsc.edu.
