# Supplementary material for: Features derived from blood pressure and intracranial pressure predict elevated intracranial pressure events in critically ill children
Source: Sci Rep. 2022 Dec 12;12:21473. doi: 10.1038/s41598-022-25169-3 (PMC9744906; doi:10.1038/s41598-022-25169-3)

**Supplementary figure 1: Line graphs of a control dataset across a 4-hour block.** **Left** – Label A is an example of “wide” artifact and label B is an example of “narrow” artifact. **Right** - Both types of artifact are removed using a two-step artifact detection algorithm without impacting remaining data.


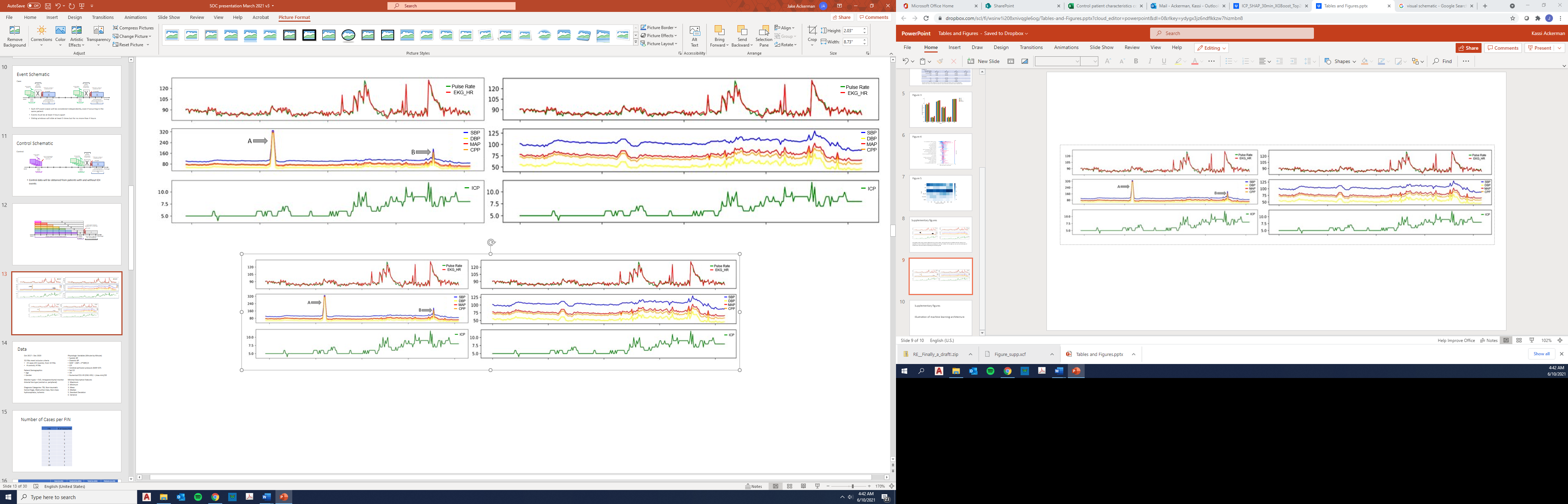


**Supplemental figure 2:** **Architectural diagram of our modeling approach**. 4-hour records from cases and controls were divided 70/30 for training and testing, respectively.  Training and testing were iterated with ten different 70/30 splits at each time window following 10 Monte Carlo simulations (bootstrapped iterations with replacement).  Extracted features at each time window were used for predictive modeling with XGBoost, random forest (RF), support vector machine (SVM), and logistic regression (LR). The optimal model from each machine learning method was tested on 10 test splits to calculate average model performance.


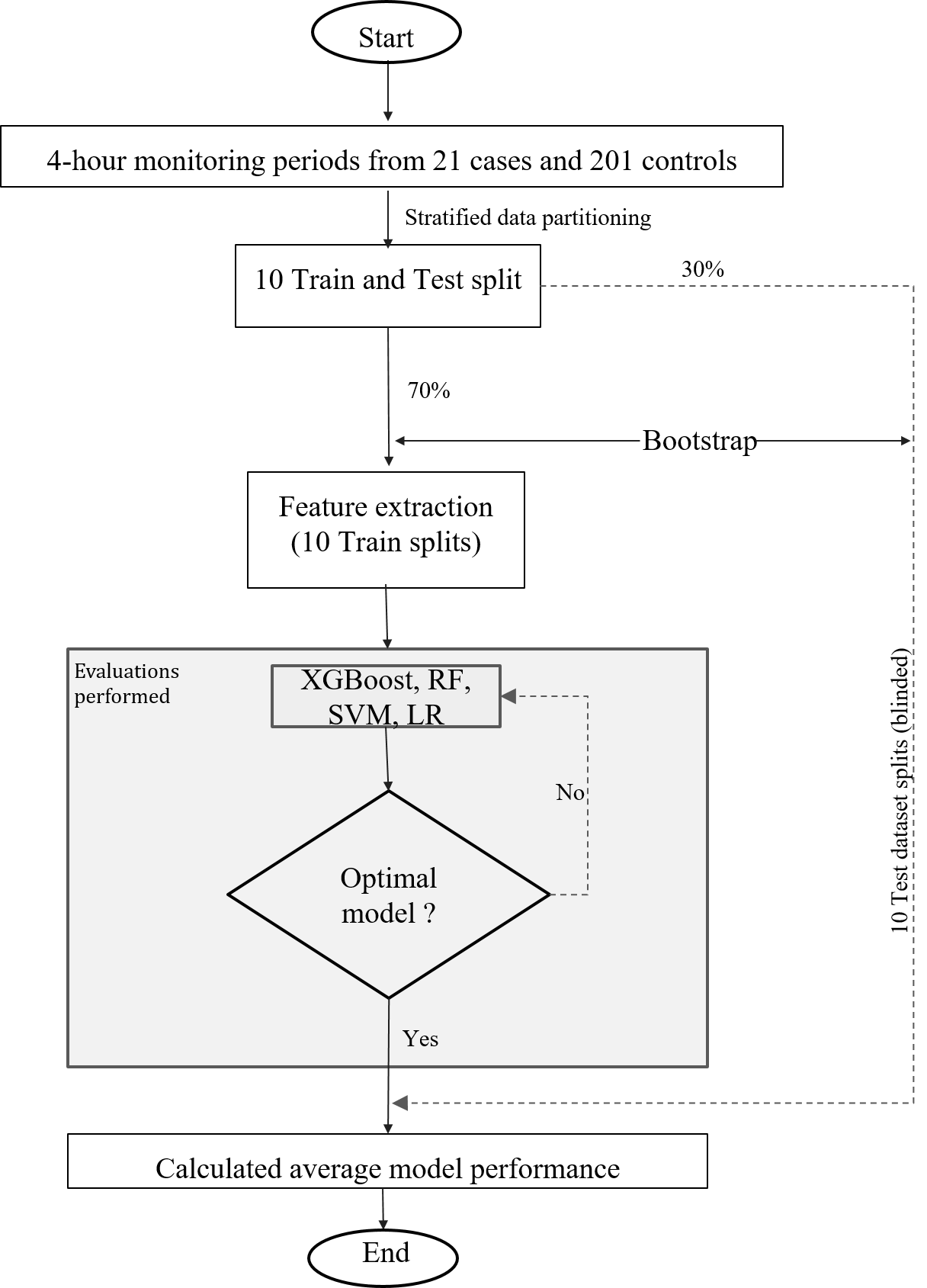


**Supplemental table 1: Clinical characteristics** by case events & control periods.

| **​** | **Case Events (n=21)​** | **Control Periods (n=200)​** | **Total (n=221)​** |
| --- | --- | --- | --- |
| Age (mean + SD)​ | 10 + 5​ | 11 + 5​ | 11 + 5​ |
| Male (%)​ | 8 (38%)​ | 76 (38%)​ | 84 (38%)​ |
| Diagnostic Category:​ | ​ | ​ | ​ |
| Traumatic Brain Injury​ | 15 (71%)​ | 126 (63%)​ | 141 (64%)​ |
| Non-Traumatic Hemorrhage​ | 4 (19%)​ | 67 (33%)​ | 71 (32%)​ |
| Obstructive Mass​ | 1 (5%)​ | 7 (3%)​ | 8 (4%)​ |
| Ischemic^§^ ​ | 1 (5%)​ | 0​ | 1 (<0.01%)​ |
| Intraparenchymal pressure monitor (%)*​ | 13 (62%)​ | 130 (63%)​ | 143 (65%)​ |
| Craniectomy (%)​ | 10 (48%)​ | 143 (71%)​ | 153 (69%)​ |
| BP Medications​ | ​ | ​ | ​ |
| Vasoactives/inotropes ​(%, Mean VIS + SD)​ | 6 ​  (29%, 3 + 5)​ | 32 ​  (16%, 1 + 4)​ | 38 ​  (17%, 1 + 4)​ |
| Anti-hypertensives​ | 6 (29%)​ | 43 (21%)​ | 49 (22%)​ |
| None​ | 9 (43%)​ | 125 (62%)​ | 134 (61%)​ |
| Pupil reactivity^†^​ | ​ | ​ | ​ |
| Both​ | 15 (71%)​ | 142 (72%)​ | 157 (72%)​ |
| One​ | 1 (5%)​ | 13 (7%)​ | 14 (6%)​ |
| Neither​ | 5 (24%)​ | 43 (21%)​ | 48 (22%)​ |
| Columns contain characteristics at the time of the record.  ^§^ Suffered a thrombotic stroke and cardiac arrest.  *Remainder had externalized ventricular drains (EVD). One patient had data from both an EVD and intraparenchymal pressure monitor but not simultaneously.  †Nearest to start of case or control periods. One patient did not have pupil exams documented due to severe ocular/facial trauma. ​ | | | |

**Supplemental Figure 3: Performance of XG Boost for predicting elevated ICP events with a 30 minute prediction horizon when model includes features from blood pressures alone.** Error bars = 95% confidence intervals.


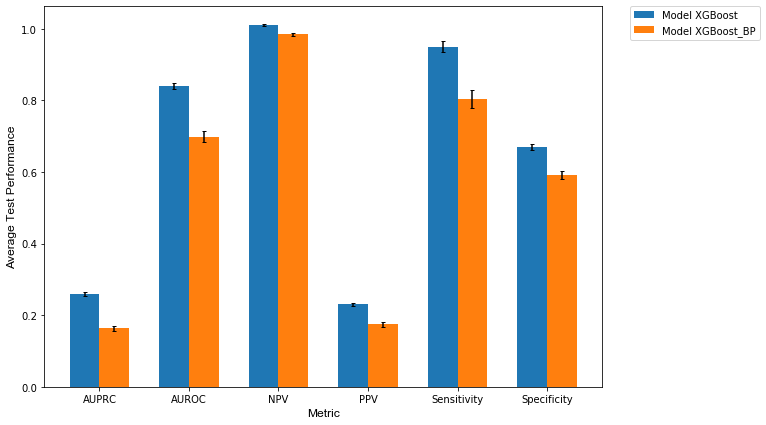


**Supplemental Figure 4: Average SHAP value plot for the XGB model which used features from the blood pressure signal only, with a 30-minute prediction horizon.** Displayed in descending order of importance are the 20 features which were most contributory along with averaged SHAP values.


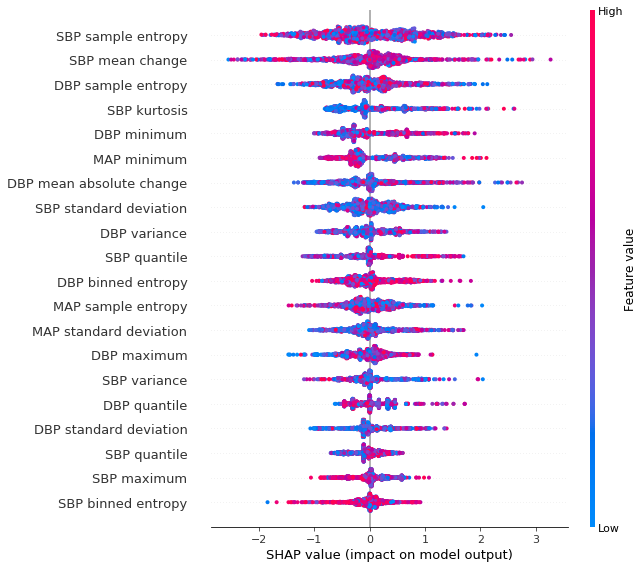

Supplement: Supplementary file 1 — Supplementary Information. [file 41598_2022_25169_MOESM1_ESM.docx]
